# Mobilizing agencies for incidence surveys on child maltreatment: successful participation in Switzerland and lessons learned

**DOI:** 10.1186/s13034-017-0211-2

**Published:** 2018-01-03

**Authors:** Andreas Jud, Céline Kosirnik, Tanja Mitrovic, Hakim Ben Salah, Etienne Fux, Jana Koehler, Rahel Portmann, René Knüsel

**Affiliations:** 1 Child and Adolescent Psychiatry/Psychotherapy, University of Ulm, Ulm, Germany; 2 School of Social Work, Lucerne University of Applied Sciences and Arts, Lucerne, Switzerland; 3 Observatory on Child Maltreatment, University of Lausanne, Lausanne, Switzerland; 4 School of Information Technology, Lucerne University of Applied Sciences and Arts, Rotkreuz, Switzerland

**Keywords:** Child maltreatment, Incidence, Administrative data, Knowledge mobilization

## Abstract

**Background:**

Many countries around the world lack data on the epidemiology of agency response to child maltreatment. They therefore lack information on how many children in need get help and protection or if children stand equal chances across regions to get services. However, it has proven difficult to commit child protection agencies to participation in incidence studies.

**Methods:**

The Optimus Study invested in a continuous collaborative effort between research and practice to develop a data collection for the first national study on the incidence of agency responses to all forms of child maltreatment in Switzerland. An innovative approach of utilizing individual agencies’ standardized data reduced work burden for participation respectably: any arbitrary excerpt of data on new cases between September 1 and November 30, 2016, could be uploaded to a secured web-based data integration platform. It was then mapped automatically to fit the study’s definitions and operationalizations.

**Results:**

This strategy has led to a largely successful participation rate of 76% of agencies in the nationwide sample. 253 agencies from the social and health sector, public child protection, and the penal sector have provided data.

**Conclusions:**

Valuing agencies context-specific knowledge and expertise instead of viewing them as mere providers of data is a precondition for representativeness of incidence data on agency responses to child maltreatment. Potential investigators of future similar studies might benefit from the lessons learned of the presented project.

## Background

There is widespread agreement that in order to make progress in the prevention and reduction of child maltreatment it is important for policy-makers and administrators to have information on its scope and characteristics [1]. The worldwide number of efforts to nationally collect administrative data on agencies’ knowledge of child maltreatment is, however, rare [1–3]. Countries’ instable financial situations are not the only contributor to blame as also many high-income countries lack a system of child maltreatment surveillance [3]. In many continental European countries, for example, there is no mandate for organizations in the child protection system to investigate and substantiate allegations of child maltreatment. Administrative data collection in these countries has so far primarily focused on the services provided. Another reason for lacking data are complex, federally organized child protection systems. Jud et al. [3] discuss reasons for lacking child maltreatment surveillance in high-income countries in detail. Anyhow, with lacking information on who enters the child protection systems, policy-makers and administrators do lack information about how to best allocate scarce resources to the ones most in need, change practices in assessment and intervention, train professionals, and reorganize systems for better responses [1]. So far, many researchers around the world have responded to this need using surveys to count the prevalence of child sexual victimization or physical maltreatment in the general population. The prevalence of psychological maltreatment and child neglect has been less intensely studied. Furthermore, general population surveys do not inform policy-makers about the services or agencies in their jurisdictions that have knowledge of (alleged) child maltreatment, and what they are doing or not doing when they encounter it. Such data is gathered trough “agency surveys” or by analyzing administrative data. For agency surveys, frontline workers provide information on their cases by completing questionnaires. All of these studies cover the respective country’s public child protection organizations, some additionally sentinels like schools or agencies in the (mental) health sector [e.g. 4, 5].

To counter the lack of data on agency responses to child maltreatment, the World Health Organization provided a toolkit for researchers [6]. It assembles the lessons learned from previous studies on agencies’ knowledge of child maltreatment. In all of theses studies, a knowledge mobilization approach has been essential for agency participation [7]. This approach does not consider agencies and frontline workers as mere informants and providers of data. Instead, they are viewed as trusted partners in a mutual relationship with researchers; their local and context-specific knowledge is valued [e.g. 8, 9]. Research staff acknowledges that child protection practice will only commit to participation if the research initiative is perceived as being both relevant and credible. Major barriers to overcome are agencies’ concerns on the confidentiality of data, concerns of being evaluated and compared, and, probably most importantly, work burden: Extra work for data collection will conflict with work time for clients or with the worker’s free time [7].

This article adds to the literature by presenting an innovative study design to counter the lack of national data on agency response to child maltreatment. It describes how a large participation rate of agencies has been reached using this approach in Switzerland, and provides lessons learned. Despite of being one of the world’s wealthiest countries, Switzerland lacks uniform and comparable data on child maltreatment incidents known to agencies. It therefore lacks data on how (frequently) and which victimized children receive support and protection [10].

### Child protection in Switzerland

The child protection and child welfare systems in Switzerland are structured according to the political principles of federalism and subsidiarity which include the goals of organizing service systems on the cantonal (provincial) level and providing services—whenever possible—at the lowest political level, the municipalities [11]. Consequently, there are 26 cantonal variations of organizing mandated and voluntary support for children in need. Further variations occur within cantons. This complexity on a relatively small scale of 8.5 million inhabitants is amplified by Switzerland’s cultural and linguistic variety of three major languages, German, French, and Italian. Three sectors are essential for child protection in Switzerland [11]:

- *Public child protection* The Swiss Civil Code empowers the child protection authorities to enact child protection orders if parents are unable or unwilling to remedy a situation of child endangerment. In most cases, they issue a general and unspecified mandate to a social worker in a specialized or general social service appointing him/her a deputy to the child. In more severe cases, the authorities can place the child in out-of-home care or finally withdraw parental custody. At a subsidiary level, child welfare services have to offer help and counseling to children and families free-of-charge. Child protection orders are only enacted if this support is not deemed sufficient to counter an endangerment.
- *Penal sector* In severe cases of child maltreatment, prosecution and conviction of the perpetrator(s) can be a part of protecting the child from further harm. This goal is accompanied by the societal or individual need for dispensing justice and convicting felonies. Penal authorities handling cases of criminally liable child maltreatment include the police forces, the agencies of prosecution, and the criminal courts plus specialized juvenile courts and juvenile prosecution organizations to enforce juvenile criminal law.
- While a huge variety of organizations offer help and support to children and families with difficulties, some public and private bodies have established *specialized agencies supporting children affected by child maltreatment*. They particularly include interdisciplinary child protection teams (in hospitals or regionally administered), private counseling centers focused on support for victims of child sexual victimization, and publicly funded victim aid agencies.

For more details and a discussion of the role of sentinel agencies, see Jud and Knüsel [11], a framework for mapping child protection agencies is suggested in Trocmé et al. [12]. Much of the debate on professionalizing and improving child protection in Switzerland still falls within these sectorial or disciplinary silos. Data collection is even more fragmented and far from being uniform or harmonized across or even within sectors. While most agencies still gather standardized information in an idiosyncratic approach for their agency, a few national efforts to collect child protection-relevant data at a national level nevertheless exist. These efforts include the federal annual reports of Police Criminal Statistics [e.g. 13, 14] and of services by victim aid agencies [15], the annual report on newly enacted and ongoing child protection orders [e.g. 16], and a national data set for cases of hospital child protection teams [e.g. 17, 18]. Agencies’ participation in the latter two is, however, not mandatory; incomplete or missing data regularly occur. An initiative aiming at sharing uniform data across sectors has been lacking so far.

## Obtaining agency participation in Switzerland: the Optimus Study^1^

The Optimus Study Switzerland addresses the paucity of incidence data on child maltreatment. A first cycle both included a population and agency survey on sexually victimized children and adolescents [19–21]. The population survey among adolescents highlighted the large amount of peer-to-peer sexual violence [20]. For the agency survey, weighted estimates indicate that 2.68 children per 1000 children in the population are reported to agencies based on an alleged incident of child sexual abuse. Unfortunately, the agency survey was bothered by low participation rates, especially in the French- and Italian-speaking parts of Switzerland [21]. Furthermore, it has been criticized that, for a child protection system, a focus on child sexual victimization is an isolated view. The different agencies and organizations not only intervene when sexual violence has been allegedly perpetrated, but as well to protect and support victims of neglect, physical and psychological violence. Multiple victimization is not the exception, but rather the rule [e.g. 22, 23].

To address these criticisms and to boost participation in a future wave of data collection, cycle 2 of the Optimus Study Switzerland reached out to stakeholders in the field of child protection—both administrators and policy-makers at the national, regional and municipal level, as well as frontline workers. The goal of this knowledge mobilization effort was to share and operationalize definitions of child maltreatment and its subtypes across sectors, to find solutions for addressing work burden for participating agencies, and creating a practice-validated and therefore relevant and credible questionnaire. It resulted in the first Swiss study on agency response to all forms of child maltreatment.

### Establishing a multisite and multidisciplinary research team

Establishing familiarity with the different linguistic, regional, and disciplinary contexts has been a first step to present child protection practitioners with a trustworthy research team. Much like in other linguistically diverse countries such as Belgium or Canada, agencies in the linguistic minority parts of Switzerland feel easily dominated by organizations representing the major language region. It has therefore been essential to locate the research team both in Lausanne (French-speaking part) and in Lucerne (German-speaking part). Furthermore, the team assembles researchers of different disciplinary backgrounds relevant to the field. Their affiliations, Observatory on Child Maltreatment at University of Lausanne and Lucerne School of Social Work, are well known for their projects and continuing education on child protection.

The team was complemented with several collaborators, e.g. in the Italian-speaking part and from the penal sector as not all linguistic regions and disciplinary backgrounds were covered. These experts in their region and field helped as facilitators of access to individual agencies and regional or federal stakeholders in the field of child protection (see “Facilitating participation” section).

### A practice-validated set of variables

Based on the assumption that practitioners are more ready to commit to participating in an epidemiological study on child protection if the variables of the data set are perceived as relevant and feasible, administrators, frontline workers and other stakeholders in child protection were invited to develop the set of study variables in a Delphi-type approach. First, a sample of agencies from different sectors were asked to provide their set of variables for standardized data entry, their definitions-in-use for child maltreatment and its subtypes. These lists of variables were then systematically compared with each other to identify uniform data elements. They were further compared to a minimum data set for child maltreatment surveillance developed in a pan-European project [24]. Next, the resulting set of variables was presented to around 50 stakeholders in child protection. In the German-speaking part of Switzerland, half-day workshops were offered in four different cities; in the more top-down organized Latin parts of Switzerland, various administrators were visited in their offices. Stakeholders discussed advantages and disadvantages of child maltreatment definitions and operationalization, commented on their priorities of including presented or additional variables in the data set, and on the feasibility of data collection. Based on this feedback, the research team created a pre-final draft of the set of variables, their definitions and operationalization that was, once again, commented by our collaborators (see “Establishing a multisite and multidisciplinary research team” section). This process resulted in 25 variables on the caseworkers (age, gender, profession, job experience), report specifications (date, source, prior report), the maltreatment incidents (type(s), onset and frequency), child characteristics (gender, age, canton of residence, disabilities, household situation, number of siblings, socioeconomic status), the perpetrators (number of perpetrators, relation to victim, age, gender), services provided, and referrals. While both researchers and practitioners agreed that it would have been important to collect information on child maltreatment severity, caregiver demographics and family risk factors, these variables were rarely available in a standardized way across sectors or operationalized too differently to map on common definitions. They could therefore not be included in this minimum data set.

### Mapping agencies’ administrative data on the study data set

**Graph 1:**
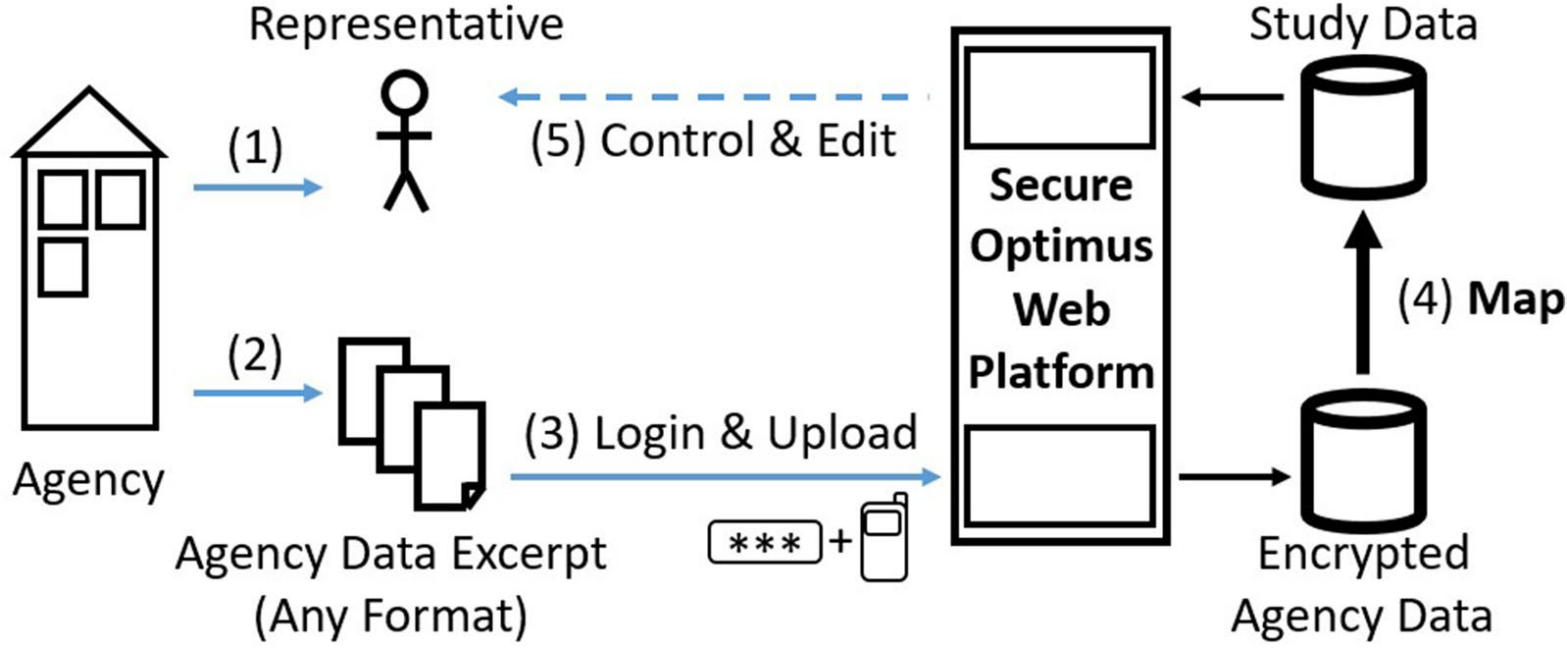
Secure workflow of data acquisition and integration

Practitioners readily embraced the idea of shared uniform data across sectors of child protection in the workshops. They however expressed concerns that the work burden of manually completing forms would decrease participation respectably and advocated for valuing agencies’ efforts of data collection. This led to an innovative approach of mapping the agencies’ pre-existing administrative data onto the study data set. We have added computer science specialists to our team who developed a procedure both guaranteeing user-friendliness and data security. Data acquisition and integration proceeded within a secure workflow (see Graph 1):

1. Each participating agency determined a representative who was registered with the web-based data integration platform.
2. In the ideal case, the representative was able to create an anonymized excerpt from the agency’s standardized data collection that corresponded with the reference period of September 1, 2016 to November 30, 2016.^2^ However, the excerpt could also contain original data covering longer periods if cantonal data protection law allowed for the transfer of such data.
3. He/she then uploaded these excerpts to the secure web-based platform using two-factor authentication (username/password and code via SMS). The study’s platform was able to anonymize and process any arbitrary format of excerpts.
4. Once uploaded, data was encrypted and removed from the web platform immediately. Algorithms mapped the agency’s individual data set onto the study data set with uniform definitions and operationalization. Variables not corresponding with the study data set were filtered out, as were any potential personal identifiers that had not already been removed before uploading. Any leftover personal identifiers were deleted immediately.
5. After mapping, agency representatives were able to complete missing data manually through a secure web interface. Most of the participating agencies lacked one or more of the study variables in their individual set of standardized administrative data. However, many agencies had information on the missing standardized data available from individual notes in the case files.

During data collection, a multilingual helpline was offered to support agencies and address all their questions. The workflow was defined within a detailed 15-page security concept and architecture document. It was established with and reviewed by the responsible ethics committee and all 26 cantonal data protection officers (plus five municipal data protection officers of large cities) to ensure conformity with ethical guidelines, federal and cantonal legislation on data protection, and to disperse any potential concern of confidentiality.

### Facilitating participation

In addition to the practice-validated data set and the innovative and timesaving approach to data collection, further steps were implemented to facilitate agencies’ participation. Several of these steps pertain to the invitation to participate: first, many agencies and stakeholders were contacted informally by our collaborators to introduce the study to their peers. Credibility and relevance of the formal invitation letter was considerably increased through support letters from the federal office responsible for coordinating child protection and several supra-cantonal organizations. To guarantee a clear, concise and non-academic style in the invitation letter, the invitation letter was reviewed both by communication experts and several stakeholders from child protection practice. The invitation letter addressed major concerns such as confidentiality of data and the concern of being evaluated or compared. To counter the latter, we have guaranteed that individual agencies will not be identified once results will be presented. Furthermore, the invitation had been sent out well in advance of data collection to allow for addressing potential concerns and all letters were addressed individually rather than just anonymously “to whom it may concern”.

If the agency did not respond to the invitation letter, we have followed-up by several telephone calls. Once an agency accepted to participate, an individual contact person was identified that would upload the excerpts from their agency’s software (see “Mapping agencies’ administrative data on the study data set” section). To further guarantee a constant exchange with agencies and other stakeholders, we have provided a biannual newsletter on the project’s progress.

For some agencies, the work burden to participate was reduced dramatically if a national data set had already been established for their type of organization (see “Child protection in Switzerland” section). They either had to give us (written) consent of accessing their data in the national data set. For the two national data sets in responsibility of the Federal Statistical Office (FSO), rights had already been transferred to the FSO, so we had access to all police and victim aid agency data via contract with the FSO. In addition, some data was directly exported and uploaded from the IT systems of a software vendor whose products are in use by a number of agency. An agency only needed to charge the vendor with the upload who then worked directly with the computer specialists of the study team. Obviously, this procedure called for a budget to reimburse the vendor.

## Participation rate of agencies in Optimus Study 3

All these different measures culminated in a largely successful agency participation rate of 76% in total, or 253 participating agencies out of 334 sampled. The population of agencies in the three essential sectors for child protection in Switzerland summed up to 545 agencies at the time of data collection. With 46% of all organizations in these three sectors, our sample of 253 participating agencies accounts for a large proportion of agencies in the Swiss child protection system.

Participation was largely comparable in the German-speaking part (78%) and in the Latin parts of Switzerland (70%). Both access to data via direct uploads of agencies individual administrative data or indirectly via access to national data sets contributed essentially to participation (see Table 1).

**Table 1:** Participating organizations by region and type of participation

	Number of agencies						
	German-speaking part			Latin parts			Grand total
	Public child protection	Penal sector^a^	Social and health sector	Public child protection	Penal sector^a^	Social and health sector	
	n (%)	n (%)	n (%)	n (%)	n (%)	n (%)	
Sample	152	22	60	51	8	41	334
Participation (total)	117 (77)	22 (100)	44 (73)	31 (61)	8 (100)	31 (76)	253 (76)
Uploading agency’s individual administrative data	69	8	18	22	6	20	143
Giving access to own data in national data set	48	14	26	9	2	11	110
Non-participation (total)	35 (23)	0 (0)	16 (27)	20 (39)	0 (0)	10 (24)	81 (24)
Do not participate to the study	29	0	12	10	0	6	57
Communication failed	6	0	4	10	0	4	24

^a^Agencies included in the penal sector are police forces

The reason for non-participation was rarely rejection. Instead, the 57 actively declining agencies did not collect standardized administrative data at all or only in a very basic way and were therefore not able to create excerpts. Another main reason for declining participation was excessive agency workload—including agencies that first accepted to participate, but later did not upload their data. Finally, 24 out of 81 non-participating agencies have been considered declining after five unsuccessful telephone calls (in different weeks at different times) to contact the agency’s director.

## Discussion

Epidemiological studies on agency responses to child maltreatment are still much needed [1]. To achieve a high agency participation rate in such a research initiative, an approach that views the child protection practice as partners instead of informants is essential, but not sufficient. Researchers have also to address work burden as a major barrier to participation. The second wave of data collection of the Optimus Study Switzerland adequately included these pillars of agency participation in their project to reach a highly satisfying overall participation rate of 76% of the sample. Advantages and caveats of the study design are discussed, so readers might be able to potentially use our procedure as an example of “good practice”.

Primarily, work burden has to be addressed as a major barrier to participation as agencies are already struggling to allocate scarce resources to the most urgent problems and many child protection workers will complain that they are overworked [7]. While producing a data export for a 3-month reference period and uploading it onto a secured web-infrastructure was indeed a timesaving way of participating in an epidemiological study for a majority of agencies, some software environments did not allow for an easy processing: The export function was restricted to a few variables or the software lacked an export function completely.

The innovative design of mapping a multitude of different administrative data formats onto the study data set (see “Mapping agencies’ administrative data on the study data set” section) not only reduced work burden for agencies, but was also a means to appreciate agencies’ previous efforts. Somewhat surprisingly, some agency representatives deemed the process of exporting data from their software as too tedious and preferred to collect data manually instead. So we additionally created an excel form with the study data set to for manual completion and easy upload onto the web-based platform. The excel form came also in handy for those small agencies that did not collect standardized administrative data at all.

Confidentiality is without any doubt an important ethical precondition for research on agency response to child maltreatment. Dealing with almost three dozen data protection officers and their feedback, however, was a time-consuming endeavor. Based on our insights into data storage of agencies, it is obvious that the security of our study data sometimes largely exceeds data security of agencies. Literacy in information technologies was at a low level for many agencies, only large agencies employ their own IT specialists. Some small agencies even had tools in use that store their data on servers in the US—outside of Swiss legislation and potentially accessible to unwanted third parties.

While it is obvious that participation will benefit from the efforts presented in this article, this procedure of knowledge mobilization is associated with an extensive temporal investment of the research team and therefore considerable budgetary resources. Our first contacts with stakeholders took place in 2012; data collection was completed in 2017. For many researchers it will be challenging to convince a scientific foundation to support a lot of exchange with participants that will not directly and/or timely lead to data and findings—we also had to invest a lot in advocating our study to our funder. Furthermore, a knowledge mobilization approach may challenge a researcher’s career goals as much of the work cannot easily be transferred into written output.

The innovative and timesaving approach is also challenged by missing data. While gender and age of the victim and the type of violence he or she suffered are available for the majority of cases, data on the perpetrator(s), child and caregiver risk factors are collected quite differently by the various agencies in different sectors—if collected at all. An implicit goal of this study was also to identify shortcomings in agencies’ individual data collection in order to define strategies towards a more uniform and shared approach to data collection on children and families in need.

## Conclusion: on the road to child maltreatment surveillance

Representatives from the relevant federal offices and supra-cantonal bodies welcomed the Optimus Study as a bottom-up initiative; administrators readily committed to the goal of shared uniform data, but perceived a lack of political will to establish a national surveillance of child maltreatment incidents. The present research initiative will identify gaps in providing support and protection to maltreated children, an especially vulnerable group of citizens. Administrators expressed their hope that the identified gaps will help convincing policy-makers to take steps towards establishing a national surveillance procedure.

Our study was also accompanied by advocacy efforts to improve the sustainability of our approach and to further pave the ground for a nationwide child maltreatment surveillance. We have reached out to policy-makers in advance of publishing our findings. An advocacy company supports and overviews all our communication activities. Dissemination efforts will comprise short presentations for individual agencies and (supra-)cantonal stakeholders, a practice-oriented research brief, press releases, etc. This strategy guarantees that dissemination of the findings not only reaches academics, but also has its impact on policy-makers so that epidemiological research can have an impact on children’s lives.

## Footnotes

1 The label “Optimus Study” encompasses projects on child sexual abuse and child maltreatment epidemiology in different countries around the world, namely in China, South Africa and Switzerland. All projects have been sponsored by the private UBS Optimus Foundation, Switzerland.

2 A 3-month reference period for data collection has both been chosen to reduce work burden and for correspondence with previous agency surveys 2 [4].
